# Hyperbaric oxygen therapy for the treatment of rectovaginal fistulas in patients with Crohn’s disease: results of the HOT-REVA pilot study

**DOI:** 10.1093/bjsopen/zrab042

**Published:** 2021-05-28

**Authors:** C A Lansdorp, C J Buskens, K B Gecse, G R A M D’Haens, R A van Hulst

**Affiliations:** 1 Department of Anaesthesiology/Hyperbaric Medicine, Amsterdam University Medical Centre, location AMC, Amsterdam, the Netherlands; 2 Department of Surgery, Amsterdam University Medical Centre, location AMC, Amsterdam, the Netherlands; 3 Department of Gastroenterology and Hepatology, Amsterdam University Medical Centre, location AMC, Amsterdam, the Netherlands

## Abstract

**Background:**

Positive effects of hyperbaric oxygen (HBO) on perianal fistulas in Crohn’s disease (CD) have been described, but the effect on rectovaginal fistulas (RVFs) has not yet been studied. The aim was to investigate the efficacy, safety and feasibility of HBO in patients with RVF in CD.

**Methods:**

In this prospective study, consecutive CD patients between November 2018 and February 2020 presenting with RVF at the outpatient fistula clinic of the Amsterdam University Medical Centre were included and selected to receive treatment with 30 daily HBO sessions, if fistulas were actively draining and any concomitant treatment regimen was stable at least 6 weeks prior to start of HBO. Patients with a stoma were excluded. The primary endpoint was clinical closure at 3-month follow-up, defined as cessation of complaints and/or closure of the external orifice if visible at baseline. Secondary outcomes were improvement of concomitant perianal fistulas as measured by the perianal disease activity index (PDAI) and fistula drainage assessment (FDA), as well as improvement in patient-reported outcomes (visual analogue scale (VAS), inflammatory bowel disease questionnaire (IBDQ), faecal incontinence quality of life scale (FIQL) and female sexual functioning index (FSFI)) at 3-month follow-up.

**Results:**

Out of 14 eligible patients, nine patients (median age 50 years) were treated, all of whom had previously had one or more unsuccessful medical and/or surgical treatments for their RVF. Clinical closure occurred in none of the patients at 3-month follow-up. There was no improvement in PDAI and patient-reported outcomes (VAS, IBDQ, FIQL and FSFI). Two patients had concomitant perianal fistulas; using FDA, one patient had a clinical response and one patient was in clinical remission 3 months after HBO. There were two treatment-related adverse events during HBO concerning claustrophobia and fatigue. Furthermore, two patients had a surgical intervention due to RVF and two patients were treated with antibiotics for a urinary tract infection during follow-up. One patient had a dose reduction of ustekinumab because of decreased luminal complaints.

**Conclusion:**

Treatment with HBO was feasible, but in this therapy-refractory cohort without deviating ostomy no clinical closure of RVF or improvement in quality of life was seen 3 months after HBO. Treatment with HBO alone in this specific group of patients therefore appears to be ineffective.

## Introduction

Fistulas are common complications of Crohn’s disease (CD), with every third CD patient having at least one fistulizing episode during his or her disease course^1^. Different types of fistulas can occur, the greatest proportion being perianal fistulas. Three to ten per cent of female patients with CD suffer from a rectovaginal fistula (RVF), making CD the second leading cause of RVF^2–4^; obstetric injuries are the leading cause.

Clinical manifestations of RVF in CD include vaginal passage of air and/or stool, recurrent vaginal infections and dyspareunia, although up to one fifth of patients are asymptomatic^4^. RVFs have a significant impact on quality of life^5^. Diagnosis of RVF can be difficult and is mostly based on clinical grounds, with inspection (if necessary, under anaesthesia), anorectal ultrasonography and MRI as additional tools^4^.

There are no evidence-based randomized controlled trials for the appropriate management of RVFs. A recent consensus of the European Crohn and Colitis Organisation (ECCO) concluded that the use of anti-tumour necrosis factor (-TNF) therapy can be beneficial in some cases, but that complete closure is rare^6^. A *post-hoc* analysis of patients with RVF who were treated with infliximab in the ACCENT II study showed closure of 60.7 and 44.8 per cent of RVFs at weeks 10 and 14, respectively^7^. The duration of RVF closure was longer in the infliximab 5 mg/kg maintenance group than in the placebo group. Surgical repair through, for example, mucosal advancement flaps (including diverting ostomy) is often attempted, but only successful in approximately half of the cases, and recurrence rates are high^6^^,^^8^. Surgical intervention should be considered with great caution, due to the low success rates and the possible increase in symptoms in case of failure. A recent trial investigated the safety and feasibility of allogenic, adipose-derived stem cells in 10 women with RVF in CD, combined with a rectal or vagina flap as deemed necessary by the treating physician. In three patients the fistulas were healed at 52-week follow-up^9^.

Hyperbaric oxygen (HBO) therapy has been reported as a potential adjunctive treatment for patients suffering from inflammatory bowel disease (IBD)^10^. HBO consists of breathing 100 per cent oxygen under higher-than-normal atmospheric pressure: usually 202–253 kPa (equivalent to 2.0–2.5 atmosphere absolute). The hyperoxygenation and oxidative stress associated with HBO have been shown to result in, amongst other changes, anti-inflammatory effects, stem cell mobilization and up-regulation of growth factors^10–12^. HBO treatment for chronic problems (e.g. wound healing) usually involves giving daily sessions for 6–8 weeks. The Undersea and Hyperbaric Medical Society, a non-profit organization which plays an important role in providing scientific and medical information on hyperbaric medicine, currently lists 14 indications for HBO therapy^13^. These include late radiation tissue injuries, diabetic foot ulcers and carbon monoxide poisoning. The therapy is generally considered safe with limited complications, with barotrauma of the ears or sinuses and transient myopia being the most common^14^.

Apart from a single case report, the use of HBO for RVFs in CD patients has not been described previously^15^. The objective of the present Hyperbaric Oxygen Therapy for the treatment of RectoVaginal fistulas in Crohn's Disease (HOT-REVA) study was to investigate changes in clinical fistula closure and quality of life after treatment with HBO, and to assess the safety and feasibility of this intervention in patients with Crohn-related RVFs.

## Materials and methods

### Study design

The HOT-REVA study was a prospective interventional study. The study was performed in accordance with the principles of the Declaration of Helsinki and Good Clinical Practice guidelines. The protocol was approved by the Medical Ethical Committee (METC 2018_248) and registered at the Dutch Trial Registry (NL6755/NTR7624).

### Setting, participants and recruitment

The study was conducted in the Amsterdam University Medical Centre (UMC), location AMC. Daily hyperbaric treatment was available in the hyperbaric unit of the Amsterdam UMC as well as in seven other hyperbaric chambers across the Netherlands. Patients were recruited at the multidisciplinary outpatient (fistula) clinic of the IBD centre. All consecutive eligible patients were invited to participate, and all participants provided written informed consent. Patients of 18 years or older with a confirmed diagnosis of Crohn’s disease with one or more rectovaginal fistula(s) (as defined by guidelines of the European Crohn’s and Colitis Organisation)^6^^,^^16^ were eligible to participate. Any concomitant treatment regimen had to be stable for at least 6 weeks prior to the start of HBO, that is, no starting of antibiotics, no surgical intervention other than seton placement or removal, no addition of immunosuppressants and/or no dosage changes of biologicals. Patients with a stoma were excluded, as well as patients who were unfit for hyperbaric treatment as assessed by the hyperbaric physician (i.e., patients with a contraindication for the therapy). Another exclusion criterion was the presence of a fluid collection/abscess related to RVF that required surgical drainage.

### Interventions

Patients were treated with 30 HBO sessions on consecutive days excluding the weekends, that is, for a duration of 6 weeks. Sessions consisted of administration of a total of 80 minutes of 100 per cent oxygen at 243–253 kPa. This treatment profile was similar to the normal clinical HBO protocol in the Netherlands and remains well within non-decompression and oxygen toxicity limits. Treatment was started directly after inclusion in the study and after investigation of fitness for hyperbaric treatment by the hyperbaric physician. All patients were treated in a multiplace chamber. If patients received less than 25 sessions of HBO or they missed more than two consecutive sessions (excluding weekends) they would be excluded and replaced, or the treatment regimen would be started anew. If patients had one or more setons in place, these were removed at the start of HBO in order to allow for fistula closure. The medical treatment regimen patients received for their CD remained unchanged in principle, but, if necessary, a change in medication could be made, also at the discretion of the treating physician.

### Outcomes

The primary outcome of the study was clinical closure of the rectovaginal fistula, defined as cessation of complaints (i.e., passage of flatus and/or stool per vagina), and/or closure of the external orifice if visible at baseline, 1 and 3 months after HBO. The secondary outcomes, assessed 1 and 3 months after HBO, were improvement of any co-existing perianal fistulas measured by the perianal disease activity index (PDAI) compared with baseline, as well as clinical response and remission as measured by the fistula drainage assessment (FDA) 3 months after HBO. According to FDA guidance, clinical response is defined as a reduction of 50 per cent or more in the number of draining perianal fistulas compared with baseline, and clinical remission defined as the absence of draining fistulas upon gentle finger compression^17^. Patient-reported outcomes were also assessed at 1 and 3 months after HBO by scores of a visual analogue scale (VAS, which records the respondent’s self-rated health on a scale from 0 to 100, with higher scores indicating better health) and the Inflammatory Bowel Disease Questionnaire (IBDQ, range of 32–224 with higher scores reflecting better quality of life)^18^. For the IBDQ, relevant clinical response is defined as an increase of 27 or more points and clinical remission as a score of at least 168^19^. Faecal incontinence quality of life scale (FIQL, consisting of 29 items in four domains with scores ranging from 1 to 4, with lower scores reflecting lower quality of life) was also assessed, as well as the minimally important change estimate (score increase of 1.1–1.2)^20^^,^^21^. Furthermore, the female sexual functioning index (FSFI, consisting of 19 items assessing difficulties and satisfaction in sexual functioning with scores ranging from 2 to 36, with lower scores reflecting lower sexual function) was reported, as well as the proportion of patients with a score of 26 or higher (reflecting normal sexual functioning)^22^^,^^23^. A validated decision regret scale (scale 0–100 with higher scores reflecting higher regret of the decision to undergo HBO) was also assessed^24^. Lastly, patients were asked to answer ‘yes’ or ‘no’ on the question if they felt their fistula(s) had improved due to HBO treatment. Furthermore, changes in use of concomitant medication, re-interventions and adverse events during treatment and at 3-month follow-up were reported. For patients who declined to participate in the study, the reasons were documented. Clinical outcomes were assessed by the treating physician of the patients (gastroenterologist and/or surgeon) at the outpatient fistula clinic. Adverse events during HBO were assessed by the supervising hyperbaric physician. None of the assessors was blinded to the intervention.

### Statistical analysis

In order to assess changes in outcomes, safety and feasibility of HBO in patients with rectovaginal fistulas, the pilot investigation aimed to include 10 patients. Outcomes are presented with descriptive statistics (numbers, scores, percentages, for unevenly distributed data accompanied with medians and/or interquartile ranges). Given the small number of patients, no statistical tests were performed.

## Results

### Patients

Screening for eligible patients took place between November 2018 and January 2020. During this period 14 consecutive eligible patients were counselled, of whom 11 patients accepted treatment with HBO. Reasons for decline were HBO interfering with personal life, worries about the luminal activity getting worse due to the intensity of the treatment and patients’ preference to treat the RVF with anti-TNF therapy. Two of the 11 patients who accepted HBO dropped out of the study prematurely and were excluded and replaced: one due to anxiety during the first session of HBO, and the other because of stress and fatigue after 17 sessions.

Due to the COVID-19 pandemic, non-emergency HBO treatment in the Netherlands was ceased and the study was stopped prematurely, with a total of nine patients being treated per protocol with HBO (instead of the intended population of 10 patients). All nine patients completed the 3-month follow-up. For one patient, the 1-month follow-up visit is missing because of hospitalization for a luminal flare-up of CD, and in another patient the patient-reported outcomes are missing at 1-month follow-up.

### Baseline characteristics

Baseline characteristics of these nine patients are shown in *Tables 1* and *2*. All patients had a previous diagnosis of RVF made by their primary physician (gastroenterologist and/or surgeon), based on a combination of clinical grounds and additional examinations, such as examination under anaesthesia and/or MRI, following current guidelines for the diagnosis of RVF^6^^,^^16^. All patients had ongoing complaints of their RVF (passage of flatus and/or stool per vagina) at baseline as a sign that the RVF was actively draining. Furthermore, in three patients the external opening of the RVF was visible during (external) physical examination. Luminal disease activity (based on the results of the last endoscopy in case of stable luminal complaints) was present in the ileum (1 patient), colon transversum (1 patient) and the rectum (1 patient). Concomitant medications that were used were combination therapy consisting of anti-TNF combined with an immunomodulator (4 patients), anti-TNF monotherapy (2 patients) and ustekinumab (2 patients). Most patients (7 of 9) had also used one or more other medical treatments in the past. One patient had had two previous surgical closure attempts under defunctioning ostomy: transperineal repair with biological mesh interposition and later full-thickness rectal advancement flap (with recurrence of complaints after restoration of continuity of the intestine). Median disease duration of the RVF was 2 years, with the longest disease duration being 22 years in one patient. Two patients had concomitant perianal fistulas: one patient with one external opening (without seton drainage) and one patient with two external openings (with two setons). There were no co-morbidities relevant to the fistulas or hyperbaric treatment.

**Table 1:** Baseline characteristics of patients undergoing hyperbaric oxygen therapy

Characteristic	Study cohort (*n* = 9)
Age (years)*	50 (40–55)
**Active smoking**	1 (11)
**Luminal disease activity present**	3 (33)
**Concomitant use of medication**	8 (89)
**Previous attempt at surgical closure**	1 (11)
Disease duration CD (years)*	7 (2–14)
Disease duration RVF (years)*	2 (1–6)
**Complaints of RVF**	
Passage of faeces per vagina	9 (100)
Passage of flatus per vagina	6 (67)
**RVF visible during physical examination**	3 (33)

Values in parentheses are percentages, unless indicated otherwise; values are median (i.q.r.). CD, Crohn’s disease; RVF, rectovaginal fistula.

**Table 2:** Characteristics of patients undergoing hyperbaric oxygen therapy

	Age (years)	Smoking status	Duration of CD (years)	Duration of RVF (years)	Concomitant medication	Previous medication	Previous surgeries	Luminal activity	Physical examination
**Case 1**	29	Previous smoker	1	1	Adalimumab, 6-mercaptopurine	None	None	Colon transversum	RVF not visible, one concomitant perianal fistula
**Case 2**	42	Never	2	1	Adalimumab, 6-thioguanine	Infliximab, budesonide	None	None	RVF visible and clearly productive
**Case 3**	51	Never	16	3	None	Prolonged antibiotics, probiotics, corticosteroids	Proctocolectomy with pouch (initial diagnosis of ulcerative colitis)	None	RVF visible
**Case 4**	56	Never	12	2	Ustekinumab, beclomethasone	Prednisone, adalimumab, 6-mercaptopurine, infliximab, anti-MAdCAM antibodies, vedolizumab	None	Rectum	RVF not visible
**Case 5**	60	Quit >6 months ago	6	7	Infliximab, azathioprine	None	Multiple surgeries for urinary incontinence, including elevate posterior and multiple tension-free vaginal tapes, with one mesh near RVF	None	RVF not visible
**Case 6**	48	Never	7	1	Ustekinumab	Infliximab, 6-mercaptopurine	Subtotal colectomy with ileosigmoideal anastomosis	Anastomosis and ileum	RVF not visible
**Case 7**	54	Never	8	6	Infliximab	6-mercaptopurine, prolonged antibiotics	Full-thickness advancement flap for perianal fistula	None	RVF visible
**Case 8**	50	Yes	22	22	Infliximab	Azathioprine	Colectomy with ileorectal anastomosis; transperineal repair with surgisis interposition; full-thickness rectal advancement flap; defunctioning ostomy	None	RVF not visible
**Case 9**	38	Never	3	2	Infliximab, azathioprine	Prolonged antibiotics	Ileocaecal resection	None	RVF not visible, two concomitant perianal fistulas

CD, Crohn’s disease; RVF, rectovaginal fistula.

### Management and adverse events

Eight patients completed 30 sessions of HBO per protocol without interruptions. One patient had an upper respiratory tract infection after 15 sessions, causing her to miss three sessions in a row. However, in collaboration with the patient and the research team, the HBO treatment was extended to a total of 40 sessions, including 25 consecutive sessions which were followed without interruption. Two patients had setons for concomitant perianal fistulas; in one patient the seton was removed before the start of HBO per protocol. For a patient that had two concomitant perianal fistulas with two seton drains, one of the drains remained *in situ* during HBO and follow-up because the fistula was deemed too productive to remove the seton by the treating surgeon. In nine patients who completed the course of HBO, two patients received antibiotics because of urinary tract infections and one patient had a dose reduction of ustekinumab (once per 4 weeks to once per 8 weeks) because of decreased luminal complaints during follow-up. Another patient stopped all concomitant medication (ustekinumab) against medical advice directly after HBO and later presented with a small-bowel obstruction for which surgical resection was performed. One patient had a surgical reintervention for her RVFs: because of perianal pain 1 month after HBO, an inspection under anaesthesia was performed. An undrained collection was found near her RVF, for which two setons were placed, creating two perianal fistulas. Another patient was scheduled for a deviating colostomy at the end of the 3-month follow-up because of ongoing complaints of her rectovaginal and perianal fistulas.

### Primary outcome: clinical fistula closure

None of the patients achieved clinical fistula closure at the 1- and/or 3-month follow-up; none of the patients reported cessation of complaints. In one patient the external vaginal orifice of the RVF that was visible at baseline was no longer identified at 3-month follow-up, but complaints of passage of flatus and gas per vagina were still present.

### Secondary outcomes

An overview of secondary outcomes per patient can be found in *Table 3*. The median PDAI score at baseline was 10 (i.q.r. 10–12). At 1- and 3-month follow-up, the median scores were 10 (i.q.r. 9–10.5) and 9 (i.q.r. 8.75–11), respectively. None of the patients had inactive disease (defined as a PDAI score of 4 or less) at follow-up^25^. Two patients had co-existing perianal fistulas at baseline. As assessed by FDA criteria, one patient had one external opening at baseline, which was closed at 1- and 3-month follow-up (only the RVF remained), and she therefore was in clinical remission with regard to her perianal fistulas. The other patient (with two openings at baseline) had one remaining external opening at follow-up, and therefore was a clinical responder. A third patient, with no co-existing perianal fistulas at baseline, had an undrained collection at 3-month follow-up, in which two setons were placed, creating two perianal fistulas.

**Table 3:** Secondary outcomes for patients who underwent hyperbaric oxygen therapy

	Time of assessment	PDAI	VAS	IBDQ	FIQL	FSFI	Comments
**Case 1**	Baseline	10	70	168	2.17	18.3	Concomitant perianal fistula clinically closed
	1-month follow-up	10	70	171	2.32	17.9	
	3-month follow-up	9	70	161	2.20	27.7	
**Case 2**	Baseline	11	93	137	2.67	23.7	Drainage of abscess during follow-up with seton placement (2×), vaginal external opening no longer visible, antibiotics for UTI
	1-month follow-up	11	95	170	2.43	24.2	
	3-month follow-up	9	57	132	1.49	22.5	
**Case 3**	Baseline	10	50	115	2.65		Meaningful improvement IBDQ, not in remission
	1-month follow-up	10	60	136	NA	NSA	
	3-month follow-up	10	70	160	2.79		
**Case 4**	Baseline	12	30	153	1.95		Dose ustekinumab decreased during HBO (4 weeks to 8 weeks interval)
	1-month follow-up	9	NA	NA	NA	NSA	
	3-month follow-up	8	70	125	171		
**Case 5**	Baseline	9	85	185	3.35		Antibiotics for UTI
	1-month follow-up	9	80	156	NA	NSA	
	3-month follow-up	9	79	192	3.20		
**Case 6**	Baseline	12	70	153	2.06		Ustekinumab stopped after HBO, surgical resection because of small bowel obstruction
	1-month follow-up	NA	NA	NA	NA	NSA	
	3-month follow-up	11	60	114	2.17		
**Case 7**	Baseline	10	50	177	3.54		
	1-month follow-up	7	74	190	3.31	NSA	
	3-month follow-up	6	79	186	3.34		
**Case 8**	Baseline	10	27	91	1.56		
	1-month follow-up	10	20	85	1.44	NSA	
	3-month follow-up	10	30	83	1.58		
**Case 9**	Baseline	11	75	157	2.83	19.5	Closure of one concomitant perianal fistula, scheduled for defunctioning ostomy at end of follow-up because of ongoing complaints of other fistulas
	1-month follow-up	10	75	166	2.89	17.9	
	3-month follow-up	11	65	152	2.56	19.7	

PDAI, perianal disease activity index; VAS, visual analogue score; IBDQ, inflammatory bowel disease questionnaire; FIQL, faecal incontinence quality of life scale; FSFI; sexual functioning index; NA, not available; NSA, not sexually active; HBO, hyperbaric oxygen therapy; UTI, urinary tract infection.

The median VAS score at baseline was 70 (i.q.r. 50–75). At 1- and 3-month follow-up, the median scores were 74.5 (i.q.r. 62.5–79) and 70 (i.q.r. 59.25–77.5), respectively.

The median IBDQ score at baseline was 155 (i.q.r. 137–173), with three patients having a score of 168 or higher indicating clinical remission. At 1- and 3-month follow-up the median scores were 161 (i.q.r. 138–171) and 155 (i.q.r. 122–167), respectively. One patient had a relevant clinical improvement as measured by IBDQ score (27 or more points difference) at 3-month follow-up, although she was still not in clinical remission.

The median total FIQL score at baseline was 2.65 (i.q.r. 2.06–2.97). The median domain scores were 2.90 for lifestyle, 2.25 for coping/behaviour, 2.56 for depression and 2.67 for embarrassment. At 1- and 3-month follow-up, the total median scores were 2.39 (i.q.r. 2.07–3.00) and 2.24 (i.q.r. 1.68–2.89). None of the patients had an improvement larger than the minimal clinically important difference of 1.1–1.2 points.

Three out of nine patients were sexually active at baseline, with a FSFI scores of 18.3, 23.7 and 19.3. At follow-up, two patients had unchanged scores, and one patient had an improvement in sexual functioning with a score of 27.7 at 3-month follow-up, indicating normal sexual functioning (cut-off score 26). None of the patients that were previously inactive became sexually active during treatment or follow-up.

The median score of the decision regret scale (range 0–100) was 20 (i.q.r. 10–30) at 1-month follow-up and 15 (i.q.r. 0–27.5) at 3-month follow-up, indicating low decision regret.

Two out of nine patients answered ‘yes’ when asked if they felt their fistula(s) had improved due to HBO treatment at 3-month follow-up.

## Discussion

In this article we presented the results of the HOT-REVA study, in which nine CD patients with RVFs were treated with HBO. Although the treatment was found to be feasible and was well tolerated, no clinical closure was seen 1 and 3 months after HBO in any of the patients. Furthermore, there was no improvement in quality of life as measured by VAS scores, IBDQ, FSFI and FIQL.

These results are discouraging, especially because HBO has been shown to have positive effects on IBD in general as well as perianal fistulas in CD^10^^,^^26^. HBO increases plasma and tissue oxygen levels, and therefore decreases hypoxia. HBO has been shown to alter signalling pathways such as hypoxia induced factor (HIF) and heme-oxygenase (HO), both involved in tissue response to hypoxia and wound repair^10^. The use of HBO in perianal fistulas has been described in several case series, showing a positive effect on fistula healing^10^^,^^15^^,^^27^. A prospective interventional trial concerning HBO for perianal fistulas has recently been executed in the Amsterdam UMC and has shown promising results, which have been submitted for publication^28^. A positive effect on perianal fistulas was also seen in the present HOT-REVA study; of the two patients with concomitant perianal fistulas at baseline, one had a clinical response and one was in remission at 3-month follow-up. The reason for failure of the treatment for RVF remains unclear. In general, RVFs are notoriously more difficult to treat than perianal fistulas, which is also reflected in lower response rates to anti-TNF therapy and allogenic, adipose-derived stem cells^7^^,^^9^. It is possible that the length of the fistula tracts (usually very short in RVF, with the diameter of the fistula exceeding the length) does not allow for adequate wound repair as one of the mechanisms of action of HBO, in contrast to the longer tracts that are seen in perianal fistulas. The ongoing pressure and space effect of faeces in the rectum in patients without deviating ostomy may also impair chances of healing in these short tracts. Furthermore, the nine patients treated in the HOT-REVA study were therapy-refractory: all patients had had an unsuccessful previous treatment with anti-TNF therapy and some patients had also had an unsuccessful surgical intervention. In some cases the RVF had already been present for a (very) long time (up to 22 years). Full epithelization of the tracts can be expected after such a long existence, which decreases the chances of success even further. Patients in the current HOT-REVA study were treated with 30 sessions of HBO therapy, and it might be possible that extending the amount of sessions would have resulted in a better effect. However, given that the concomitant perianal fistulas in these patients did respond after 30 sessions (and the RVF did not), this is unlikely.

The use of HBO for RVFs has been reported before in a 32-year-old patient with a single, RVF and active luminal disease in the rectum^15^. She was treated with 21 daily sessions of HBO, but was also started at the same time on anti-mycobacterium avium paratuberculosis (MAP) therapy. After 3 months, complete rectal and vaginal fistula healing was observed on colonoscopy and MRI, with cessation of complaints of RVF. However, after stopping the MAP therapy during follow-up, the fistula reopened and symptoms recurred.

While performing the HOT-REVA study, a patient who was excluded because of a deviating colostomy was treated off-label with 40 sessions of HBO in the Amsterdam UMC^29^. She had an RVF as well as multiple perianal fistulas, and had been therapy refractory to infliximab and the colostomy for 6 years. Three months after the treatment she had complete clinical closure of all fistulas (including the RVF). MRI showed a fibrotic fistula complex, confirming complete healing of the fistula tracts.

Although the HOT-REVA study did not show improvement in therapy-refractory patients without deviating ostomy, the previously mentioned reports and mechanisms of action give reason to believe that HBO treatment might still be effective in other cohorts of patients with RVF. Future research could involve patients with new fistulas or with a deviating ostomy, or patients receiving pre- and postoperative HBO treatment around surgical correction of their RVF.

There are several strengths and weaknesses to this study. The study was performed prospectively, with clearly defined inclusion and exclusion criteria and outcomes. The use of a 3-month follow-up allowed for detection of any late effects of HBO therapy. However, the number of patients who were included was small, and there were no controls in the study. The certainty with which conclusions can be drawn about the non-efficacy of HBO is further decreased by the heterogeneity of the study population. One patient had a mesh close to the RVF, possibly limiting treatment success, and another patient had a pouch due to a previous diagnosis of ulcerative colitis (which was later changed to Crohn’s disease). Only clinical outcomes were used to assess the effect of HBO treatment; the addition of an imaging outcome, such as MRI, could have been beneficial to exclude bias. However, large inter-rater variability in the assessment of RVF on MRI was found previously, and MRI is not always sensitive enough to detect smaller tracts^30^^,^^31^. Since no adequate objective outcome measure is available, it was decided to use clinical outcomes only, which is also in concordance with the outcome measures used in the ACCENT II trial^7^.

Finally, no clinical closure or improvement in quality of life was seen after treatment with HBO in this cohort of CD patients with therapy-refractory RVF without deviating colostomy, and treatment with HBO alone in these patients appeared to be ineffective.
